# How knowledge of the gastrointestinal absorption of elements could be used to predict transfer to milk

**DOI:** 10.1038/srep37041

**Published:** 2016-11-15

**Authors:** Brenda J. Howard, Claire Wells, Catherine L. Barnett, Steve C. Sheppard

**Affiliations:** 1 Centre for Ecology and Hydrology, Lancaster Environment Centre, Bailrigg, Lancaster, LA1 4AP, UK; 2 ECOMatters Inc., PO Box 430, Pinawa, Manitoba, R0E1L0, Canada

## Abstract

The quality and quantity of data used to derive transfer parameter values for milk are variable and there are many data gaps for elements/radionuclides which may need to be considered for risk assessment of the agricultural foodchain. There has been a recent focus on critically evaluating current methods to fill data gaps and on identifying extrapolation methods to derive suitable values for the elements, and particularly radioisotopes, with no or sparse data. The relationship between fractional absorption of elements in the ruminant gastrointestinal tract and transfer to milk has been explored to determine whether knowledge of the former can be used to predict the latter. A relationship has been derived between fractional absorption of elements and two empirical ratios commonly used to quantify transfer to milk; transfer coefficients (element concentration in milk divided by element daily intake) and concentrations ratios (concentration in milk divided by concentration in feed). We propose that fractional absorption may be used to predict the order of magnitude of the transfer to milk of elements/radionuclides for which no relevant data have yet been identified or collated.

For many years, estimates of the transfer of radionuclides to animal products has been achieved by the use of transfer coefficients defined as the activity concentration in milk (F_m_) or meat (F_f_) (Bq kg^−1^ fresh weight (fw) divided by the daily intake of the radionuclide (Bq d^−1^)) assumed to be at steady state. More recently, the use of concentration ratios (CR) has been suggested, which removes the need to quantify the total intake of the animal as it uses the concentration in the feed (Bq kg^−1^ dry weight (dw)) as the denominator rather than the intake^1^,^2^. Both these authors suggested that there is a potential advantage of the use of CR values in that they are less variable than transfer coefficients and easier to obtain.

The major sources listing F_m_ and CR values for different elements with radioisotopes have changed over the decades. In the 1960–80s, reviews by Ng^3^,^4^,^5^,^6^,^7^,^8^,^9^ were often used. In the 1990s the International Atomic Energy Agency (IAEA) published compilations of transfer parameter values for the human foodchain which included F_m_ and F_f_ values for a range of animal products in TRS 364^10^ and NCRP^11^ also published values used in a screening model. In 2009, a revised compilation of IAEA values, accompanied by values for CR, was provided in TRS 472^12^ (accompanied by Tecdoc 1616^13^ and papers^1^,^14^ which gave more detailed information of each set of values given in the TRS).

TRS 472 is based largely on original data sources for radioisotopes and stable elements, rather than reviews which were an important data source for the earlier TRS 364. Although many data are available for the three elements with important dose-forming radionuclides: Cs, Sr and I, there are many data gaps and no or little data yet identified from many other elements including natural radioisotopes.

Many compilations of data on the transfer of radionuclides to milk fill in data gaps by referring to the previous reviews or literature compilations mentioned above. However, there are significant disadvantages in the use of such approaches including a lack of rigour, provenance, quality control or peer review^15^.

In the absence of data a number of extrapolation methods are frequently used for both the human foodchain and for quantifying transfer to wildlife. These include the use of analogue elements, use of analogue animal products (e.g. sheep milk or goat milk for cow milk), Bayesian statistics and biokinetic modelling. For wildlife assessments, new approaches for extrapolation have been explored including using generic (trans-species) CR, allometry and REML (Residual Maximum Likelihood) modelling which have potential to improve prediction of radionuclide transfer by making better use of existing parameter values^17^.

Less attention has been given to extrapolation for the human foodchain, although some of the approaches discussed above^17^ could be relevant. In this paper we test a possible relationship between fractional absorption and transfer to milk that might allow a new extrapolation technique to be developed for milk. For an element to be present in milk, it first has to pass through the wall of the gastrointestinal tract. Therefore, it is reasonable to consider whether data on the fractional absorption (the fraction present in the gut that passes into the blood), which varies considerably between different elements, may be extrapolated to derive values for transfer parameters for milk. The aim of this study was to test the hypothesis that there is a relationship between fractional absorption and the transfer of elements to milk.

## Results

The method used to define the data for the ruminant absorption values differed from that of the ICRP^18^ for the human alimentary tract model where F_a_ values were based on expert review of literature. However, the F_a_ values for ruminants (Table 1) are generally within an order of magnitude of those reported by ICRP, with some exceptions (see below).

There are far more transfer parameter values for different elements available for cow milk than for goat milk. The elements with the most transfer parameter values for cow milk, with n ≥ 100, are Cs, Sr and I. For goat milk the data are less numerous with n = 27, 23 and 21 respectively for Cs, Sr and I, and ten elements with n ≤ 2 data values (Table 2).

**Figure 1:**
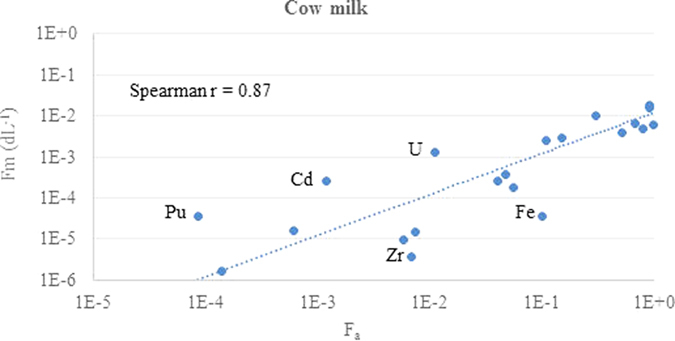
Comparison of the TRS 472 ruminant absorption values with F_m_ for cow milk using the updated animal dataset.

**Figure 2:**
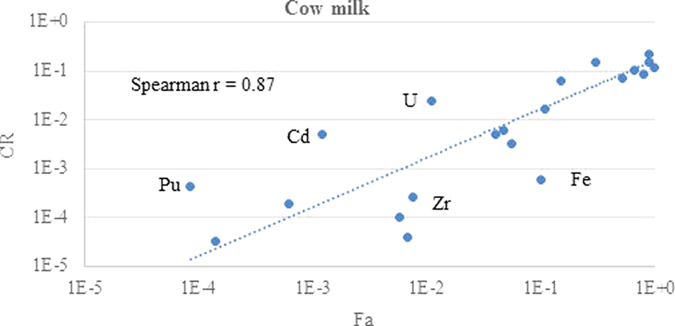
Comparison of the TRS 472 ruminant absorption values with CR for cow milk using the updated animal dataset.

**Figure 3:**
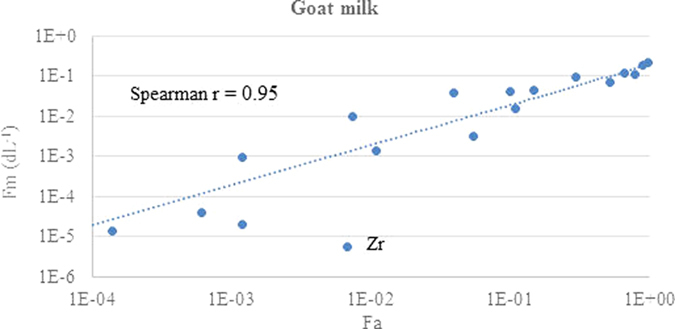
Comparison of the TRS 472 ruminant absorption values with F_m_ for goat milk using the updated animal dataset.

**Figure 4:**
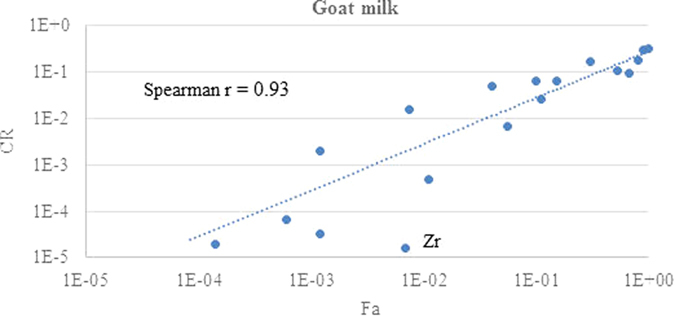
Comparison of the TRS 472 ruminant absorption values with CR for goat milk using the updated animal dataset.

The relationship between ruminant fractional absorption and transfer parameter GM values for different elements and both milk products are shown in log-log plots in Figs 1, 2, 3 and 4. Each data point corresponds to a different element.

## Discussion

The chemical form and extent of binding to soil adhered to ingested material will impact on fractional absorption by ruminants and vary between elements^16^. Nevertheless, there is a good correlation between the fractional absorption values for each element and the transfer parameter values for both milk products. The r values are similar for both F_m_ and CR in each milk product and are slightly stronger for goat milk than for cow milk. However, for both milk products there are elements that are outliers and are discussed below. In some cases possible reasons for the discrepancy can be suggested whereas for others the paucity of data makes this difficult.

There are a limited number of ruminant fractional absorption values for most elements and fewer than that for the element transfer parameter values for both milk and products. Consideration of the F_a_ values can take into account those derived by ICRP^18^ for human assessment bearing in mind that the ICRP values are derived by an expert review process and, as for the ruminant dataset, the quality, quantity and relevance of available data for each element varies. An equivalent, independent, recent, compiled literature source is not available with which we can evaluate the transfer parameter data.

For cow milk, Pu is the most obvious outlier with higher transfer parameter value than that expected from the F_a_ value. The Fa value was based on three data sources^19^,^20^,^21^. Beresford reported a derived F_a_ of 1.2E-4 from data reported for lambs fed vegetation contaminated by marine discharges from the Sellafield reprocessing plant^20^. Lower F_a_ values of 7.9E-5 and 6.5E-5 were reported for experiments in which ^239/240^Pu was administered orally in two soils, one artificially contaminated and one contaminated by Sellafield discharges^21^. These absorption values are in agreement with a range of E-4–E-5 in animals for non-oxide forms and 6E-5 to 6E-4 for organic forms reported by ICRP^18^. The transfer parameter values are based on three disparate F_m_ values of 5E-4^22^, 7.5E-6^23^ and 1.3E-5^24^. In TRS 472, a value for F_m_ of 1E-5 was given based on consideration of available data^23^, partly because the compiled data for Pu F_m_ was highly variable and there were a number of problems associated with the data available, including faecal and soil contamination of vegetation and milk samples, and the lack of equilibrium after single administration studies and especially for short duration studies. It is highly unlikely that equilibrium will ever to be achieved in agricultural animals given the long biological half-life, partly due to accumulation in bone and liver^23^. The F_m_ value for Am, an analogue for Pu, is 1.6E-6 and 1.4E-5 for cow and goat milk respectively and is not an outlier.

The Cd transfer parameter values for cow milk are higher than that expected from the F_a_ value. The F_a_ value for Cd is related to solubility in the digestive tract and high dietary levels of Ca, Cr, Mg or Zn which decrease Cd absorption^25^. The mammary gland is thought to limit Cd transport as the concentration of Cd in milk is not increased by high dietary concentrations of Cd^26^. The F_a_ value for ruminants of 1.2E-3 (Table 1) which is based on data for ^109^CdCl_2_ orally administered to a three cows^27^ is more than an order of magnitude lower than that recommended in ICRP^28^ of 5.0E-2 for all inorganic compounds of Cd based on F_a_ values reported in mice, rats, and goats. In the more recent ICRP report no F_a_ values for Cd were reported^29^. The thirteen F_m_ and CR values for Cd varied substantially over several orders of magnitude (e.g. CR - 2.7E-5 to 1.6E-1) and eleven values were derived using stable Cd^30^,^31^,^32^,^33^,^34^,^35^,^36^.

The U transfer parameter values for cow milk are higher than that expected from the F_a_ value. ICRP^18^, noted that fasting can affect uranium absorption, gives an F_a_ value of 2.0E-2 based on data for humans, which is the same order of magnitude as that for ruminants of 1.1E-2^37^,^38^ (Table 1). The seven derived transfer parameter values for U^37^,^38^,^39^,^40^ most cows were fed plant incorporated ^238^U or ^234^U and had CR values ranging from 2.4E-2 to 6.1E-2. In one study where UO_2_(NO_3_)_2_ was administered the CR was lower at 5E-3^40^.

The Fe transfer parameter values for cow milk are lower than that expected from the F_a_ value. Fe is an essential element with absorption affected by many factors^25^ including diet composition, amount and chemical form ingested (as soluble forms are better absorbed better than insoluble), animal age, conditions within the gastro-intestinal tract, requirement and body iron stores, animal health and the stage of lactation. A recommended value for F_a_ of 1.0E-1 based on data for humans^29^, which is the same value as that for ruminants^12^ (Table 1) which is based on stable element data^26^. Other reported F_a_ values for ^59^Fe given as a chloride to ruminants vary considerably from 5E-3 to 9.7E-1 in cows^41^,^42^. The Fe content of milk also varies with species and stage of lactation^25^. The transfer parameter values for Fe to milk only varied within one order of magnitude.

There are fewer obvious outliers for goat milk. The goat milk dataset has a considerably lower F_m_ and CR for Zr than would be expected from the F_a_ value. There is only a single value for Zr from both absorption and transfer to milk. The single ruminant F_a_ value of 6.8E-3 (Table 1) is based upon an experiment with 5 cows given ZrCl_2_^40^, with only brief experimental details in the review of Russian language studies transfer to milk^43^. The ruminant value is similar to that given by ICRP^18^ of 1.0E-2. ICRP discuss data for Zr of 3E-4 to 2E-3 in rats^44^ and 1E-3 and 3.5E-4 to juvenile and adult rats respectively^45^. Considerably higher absorption has been noted in younger animals due to transient uptake into the walls of the gastrointestinal tract, leading to a lower assumed Fa value of 2E-3 for all compounds of Zr^46^. The milk transfer F_m_ and CR value is based on an experiment^47^ in which three goats are given a single oral administration of ^95^Zr(IV) oxalate in a gelatine capsule. The cow milk transfer value for Zr is also relatively low at 4E-6 based on six values from dairy cows. A further problem with Zr is that analytical methods used for its determination were often inaccurate in earlier studies due to leading to wide range of reported values. Given the currently available data for Zr it is difficult to have much confidence in the currently compiled values for F_a_, F_m_ and CR and, therefore, the data for Zr used our analysis.

The approach used here for milk may also be applicable for meat. This will be explored in a subsequent analysis following the ongoing revision of the TRS 472 dataset.

We cannot expect all elements to accumulate in milk according to the relationship described here due to the metabolic factors such as homeostasis, elemental antagonism/interaction, and variation in the importance of a range of macro elements in metabolism animal age, stage of lactation, pregnancy, and biological half lives in tissues and regulation of some elements by the mammary gland. However, the potential benefit of the application of the relationship described is that if there is information on gut absorption for an element then it will enable an order of magnitude estimation of the transfer parameter values for milk for the many elements for which we have no, or little data at present. Furthermore, the approach would be consistent for different elements rather than the current situation where the method used to fill data gaps for assessments varies considerably and is of variable quality and provenance.

## Materials and Method

For TRS 472, the recommended values were derived from data compiled according to procedures agreed in the EMRAS programme of the IAEA. The data used for this comparison were in separate datasets for fractional absorption and transfer to milk.

The fractional absorption dataset^16^ included values from (i) literature studies, (ii) a then recent review of Russian-language information on ruminant absorption^43^ and (iii) an agricultural review by the National Research Council^26^. The latter source was used for the fractional absorption values of Ca, Cl, Fe, Mn, Na, P, Se and Zn. The ruminant fractional absorption values (F_a_) used for this study were reported in TRS 472^12^ and a review paper for ruminants^16^ (Table 1). For most elements there were fewer than 10 data values in the dataset.

A dataset for the transfer of a wide range of elements to milk was previously used to derive the transfer parameter values in the IAEA handbook TRS 472^12^ and associated Tecdoc 1616^13^. The revision of the dataset initially focused on updating the goat^15^ and cow milk datasets. The approaches for revising the milk datasets include a rigorous quality control (QC) of the original dataset and modification of the Russian-language data to be consistent with a review paper on radionuclide transfer to milk^48^. Further data were added through a limited literature review, including additional stable element values and derivation of additional CR values from F_m_ data using assumptions of the dry matter intakes of the animals^15^. Priority was given to updating the transfer values to cow milk for those elements for which we have fractional absorption values (Table 1) to enable a comparison to be made. Key sources of new information are now incorporated into the milk dataset^39^,^49^,^50^,^51^,^52^,^53^,^54^,^55^. Information on other adjustments made during the revision process, including the removal of data originally included in the TRS 472 dataset, will be provided in a subsequent paper on transfer parameters for elements to cow milk.

The relationship between the GM values for the F_a_ and that of F_m_ or CR was quantified using a Spearman correlation coefficient analysis, with the r values shown in the figures. The Spearman coefficient is non-parametric, based on rank scores, and was considered appropriate because it is not influenced by the log-normal tendency of these parameters and because consistent ranking of the elements is core to the hypothesis of this study.

### Data Availability

The data described here have a Digital Object Identifier doi.org/10.5285/7713d170-f6a3-4aa7-83c8-fe91278517ce and are freely available from the CEH Environmental Information Data Centre (http://eidc.ceh.ac.uk/) under the Open Government Licence. These must be referenced fully for every use of the data as: Howard, B.J., Wells, C., Barnett, C.L., Sheppard, S. (2016). Transfer of radionuclides to cow and goat milk. NERC-Environmental Information Data Centre doi.org/10.5285/7713d170-f6a3-4aa7-83c8-fe91278517ce and where appropriate the source references as cited above. Supporting information to aid in the reuse of this data is available from the EIDC.

## Additional Information

**How to cite this article**: Howard, B. J. *et al*. How knowledge of the gastrointestinal absorption of elements could be used to predict transfer to milk. *Sci. Rep.* **6**, 37041; doi: 10.1038/srep37041 (2016).

**Publisher’s note:** Springer Nature remains neutral with regard to jurisdictional claims in published maps and institutional affiliations.

## Figures and Tables

**Table 1:** Fractional absorption* for ruminants^12^,^13^,^16^.

Element	F_a_	N	Element	F_a_	N
Ag	5.6E-2	1	Na	9.0E-1	—
Am	1.4E-4	2	P	6.7E-1	—
Ba	5.5E-2	2	Pb	4.0E-2	9
Ca	3.0E-1	—	Pu	8.5E-5	3
Cd	1.2E-3	1	Ru	5.8E-3	6
Ce	6.1E-4	5	Se	5.2E-1	—
Cl	9.0E-1	—	Sr	1.1E-1	21
Co	4.7E-2	9	U	1.1E-2	2
Cs	8.0E-1	14	Y	1.2E-3	2
Fe	1.0E-1	—	Zn	1.5E-1	—
I	9.8E-1	13	Zr	6.8E-3	1
Mn	7.5E-3	—			

^*^Geometric mean unless n < 3 when arithmetic mean is given.

**Table 2:** Transfer coefficient and CR values (geometric means and standard deviations) for cow and goat milk^15^ in the MODARIA 2016 dataset for animal products for elements with F_a_ values. n/a - no data available.

Element	Cow Milk					Goat Milk				
	F_m_ GM	F_m_ GSD	CR GM	CR GSD	N	F_m_ GM	F_m_ GSD	CR GM	CR GSD	N
Am	1.6E-6	12.6	7.7E-6	1.4	2	1.4E-5	6.4	1.9E-5	8.0	2
Ba	1.8E-4	2.7	3.2E-3	3.7	17	3.1E-3	1.8	6.7E-3	2.8	2
Ca	9.9E-3	1.6	1.5E-1	1.7	15	9.3E-2	2.1	1.7E-1	2.6	11
Cd	2.6E-4	16.2	5.1E-3	17.0	13	9.6E-4	4.9	1.9E-3	4.4	4
Ce	1.5E-5	6.7	1.9E-4	8.4	8	4.0E-5	—	6.4E-5	—	1
Cl	1.8E-2	2.8	1.5E-1	2.6	3	n/a	—	n/a	—	—
Co	3.2E-4	9.2	5.8E-3	10.5	16	n/a	—	n/a	—	—
Cs	4.9E-3	2.1	8.6E-2	2.1	278	1.1E-1	2.1	1.8E-1	2.0	27
Fe	3.7E-5	3.8	5.8E-4	4.5	13	4.0E-2	—	6.4E-2	—	1
I	6.1E-3	2.7	1.1E-1	3.2	100	2.1E-1	3.0	3.2E-1	3.1	21
Mn	1.4E-5	4.9	2.7E-4	4.8	16	9.8E-3	14.8	1.6E-2	14.8	2
Na	1.6E-2	1.7	2.3E-1	1.9	9	1.8E-1	2.5	2.9E-1	2.5	3
P	1.3E-2	1.2	2.4E-1	1.4	4	1.2E-1	3.0	9.4E-2	8.2	2
Pb	3.2E-4	2.7	6.9E-3	2.6	19	3.7E-2	—	4.8E-2	—	1
Pu	3.6E-5	9.8	4.3E-4	9.6	3	n/a	—	n/a	—	—
Ru	9.4E-6	8.5	1.0E-4	7.4	6	n/a	—	n/a	—	—
Se	3.8E-3	2.1	6.3E-2	2.3	27	6.8E-2	1.5	1.0E-1	1.5	4
Sr	2.5E-3	1.7	1.8E-2	1.7	116	1.5E-2	2.0	2.6E-2	2.1	21
U	1.3E-3	2.2	2.5E-2	2.2	7	1.4E-3	—	4.8E-4	—	1
Y	n/a	—	n/a	—	—	2.0E-5	—	3.2E-5	—	1
Zn	2.8E-3	2.5	6.1E-2	1.5	18	4.3E-2	1.6	6.5E-2	1.8	7
Zr	3.6E-6	4.3	4.1E-5	3.6	6	5.5E-6	—	1.7E-5	—	1

## References

[b1] Howard B. J., Beresford N. A., Barnett C. L. & Fesenko S. Quantifying the transfer of radionuclides to food products from domestic farm animals. J. Environ. Radioact. 100, 767–773 (2009a). 19362760 10.1016/j.jenvrad.2009.03.010

[b2] Sheppard S. C., Long J. M. & Sanipelli B. Verification of radionuclide transfer factors to domestic-animal food products, using indigenous elements and with emphasis on iodine. J. Environ. Radioact. 101, 895–901 (2010). 20621399 10.1016/j.jenvrad.2010.06.002

[b3] Ng Y. C. . Prediction of the maximum dosage to man from the fallout of nuclear devices. In Handbook for Estimating the maximum internal dose from radionuclides released to the biosphere. UCRL-50163, Pt. IV. Lawrence Radiation Laboratory (1968).

[b4] Ng Y. C., Colsher C. S., Quinn D. J. & Thompson S. E. Transfer coefficients for the prediction of the dose to man via the forage-cow-milk pathway from radionuclides released to the biosphere. Lawrence Livermore Laboratory Report UCRL-51939. Lawrence Livermore National Laboratory (1977).

[b5] Ng Y. C. . Methodology for assessing dose commitment to individuals and to the population from ingestion of terrestrial foods contaminated by emissions from a nuclear fuel reprocessing plant at the Savannah River plant. Lawrence Livermore Laboratory Report UCID-17743. Lawrence Livermore National Laboratory (1978).

[b6] Ng Y. C., Colsher C. S. & Thompson S. E. Transfer coefficients for terrestrial foodchains their derivation and limitations In (ed. Kellermann H. J.), Radioaktivitat and Umwelt. Proceedings of the 12th Annual Conference of the Fachverband fur Strahlenchutz, Norderney, West Germany, 2–6 October 1978. Band I, pp. 455–481 (1979a).

[b7] Ng Y. C., Colsher C. S. & Thompson S. E. Transfer factors for assessing the dose from radionuclides in agricultural products In Biological Implications of Radionuclides Released from Nuclear Industries, Vol. II. Proceedings of an International Symposium on Biological Implications of Radionuclides Released from Nuclear Industries, Vienna, March 1979. pp. 295–318 (IAEA-sm-237/54) (1979b).

[b8] Ng Y. C., Colsher C. S. & Thompson S. E. Transfer coefficients for assessing the dose from radionuclides in meat and eggs NUREG/CR-2976, UCID-19464. (Lawrence Livermore National Laboratory (1982).

[b9] Ng Y. C. A review of transfer factors for assessing the dose from radionuclides in agricultural products. Nucl. Saf. 23, 57–71 (1982).

[b10] IAEA. Handbook of parameter values for the prediction of radionuclide transfer in temperate environments. Technical reports Series No. 364. IAEA: Vienna (1994).

[b11] National Council on Radiation Protection and Measurements, Screening Models for Releases of Radionuclides to Atmosphere, Surface Water, and Ground, Report 123 I. NCRP, Bethesda, MD (1996).

[b12] International Atomic Energy Agency. Handbook of Parameter Values for the Prediction of Radionuclide Transfer in Terrestrial and Freshwater Environment. (Technical Reports Series No. 472. IAEA, Vienna, 2010).

[b13] International Atomic Energy Agency. Quantification of Radionuclide Transfer in Terrestrial and Freshwater Environments for Radiological Assessments. (IAEA-TECDOC-1616. IAEA, Vienna, 2009).

[b14] Howard B. J., Beresford N. A., Barnett C. L. & Fesenko S. Radionuclide transfer to animal products: revised recommended transfer coefficient values. J. Environ. Radioact. 100, 263–273 (2009b). 19200625 10.1016/j.jenvrad.2008.12.015

[b15] Howard B. J., Wells C. & Barnett C. L. Improving the quantity, quality and transparency of data used to derive radionuclide transfer parameters for animal products. 1. Goat milk. J. Environ. Radioact. 154, 34–42 (2016). 26845198 10.1016/j.jenvrad.2016.01.009

[b16] Howard B. J., Beresford N. A., Barnett C. L. & Fesenko S. Gastrointestinal fractional absorption of radionuclides in adult domestic ruminants. J. Environ. Radioact. 100, 1069–1078 (2009c). 19477565 10.1016/j.jenvrad.2009.03.023

[b17] Beresford N. A. . Making the most of what we have: application of extrapolation approaches in radioecological wildlife transfer models. J. Environ. Radioact. 151, 373–386 (2016). 25850783 10.1016/j.jenvrad.2015.03.022

[b18] International Commission on Radiological Protection. Human alimentary tract model for radiological protection. ICRP Publication 100 Ann. ICRP 36 (1–2) (2006). 10.1016/j.icrp.2006.03.004 17188183

[b19] Beresford N. A. . The importance of source-dependent bioavailability in determining the transfer of ingested radionuclides to ruminant-derived food products. Environ. Sci. Technol. 34, 4455–4462 (2000).

[b20] Ham G. J., Harrison J. D., Popplewell D. S. & Curtis E. J. C. The distribution of Cs-137 plutonium and americium in sheep. Sci. Total Environ. 85, 235–244 (1989). 2814451 10.1016/0048-9697(89)90322-7

[b21] Cooke A. I. . Transfer of radiocaesium, plutonium and americium to sheep after ingestion of contaminated soil. In Prost R. (ed.), Contaminated Soils. CD:\Data\comminuic\082.PDF; CD-Rom. INRA, Paris (1997).

[b22] Sirotkin A. N. Radionuclide uptake to animal stuffs. Sel’skohozyaistvenaya radioecologiya, (eds. Alexakhin R. M. & Korneyev N. A.) 106–115 (In Russian) (Ecologiya, Moscow, 1991).

[b23] Howard B. J. . The transfer of ^239/240^Pu to cow milk. J. Environ. Radioact. 98, 191–204 (2007). 17825461 10.1016/j.jenvrad.2007.01.032

[b24] Green N., Wilkins B. T., Davidson M. F. & Hammond D. J. The transfer of plutonium, americium and technetium along the soil-pasture-cow pathway in an area of land reclaimed from the sea. J. Environ. Radioact. 27, 35–47 (1995).

[b25] National Research Council. Mineral Tolerance of animals (Second revised Edition) National Research Council of the National Academies, Washington, DC, National Academy Press (2005).

[b26] National Research Council. Nutrient Requirements of Dairy Cattle, seventh ed. National Academic Press, Washington, DC (2001).

[b27] Crout. N. M. J. . The transfer of ^73^As, ^109^Cd and ^203^Hg to the milk and tissues of dairy cattle. J. Agric. Sci. Camb. 142, 203–212 (2004).

[b28] International Commission on Radiological Protection. Limits for intakes of radionuclides by workers. ICRP Publication 30 (Part 2). Ann. ICRP 4 (3–4) (1980). 7458091

[b29] International Commission on Radiological Protection. Human alimentary tract model for radiological protection. ICRP Publication 100 Ann. ICRP 36 (1–2) (2006). 10.1016/j.icrp.2006.03.004 17188183

[b30] Nelmes A. J., Buxton R. St. J., Fairweather, & Martin A. E. Implication of the transfer of trace metals from sewage sludge to man. Trace substances in environmental health. (ed. Hemphill D. D.) 8, 145–153 (Columbia, Missouri, Science Reviews Ltd., Northwood, USA, 1974).

[b31] Van Bruwaene R., Gerber G. B., Kirchmann R. & Colard J. Transfer and distribution of radioactive cadmium in dairy cows. Int. J. Environ. Stud. 19, 47–51 (1982).

[b32] Sharma R. P., Street J. C., Shupe J. L. & Bourcier D. R. Accumulation and depletion of cadmium and lead in tissues and milk of lactating cows fed small amounts of these metals. J. Dairy Sci. 65, 972–979 (1982). 7108012 10.3168/jds.S0022-0302(82)82298-4

[b33] Fitzgerald P. R., Peterson J. & Lue-Hing C. Heavy metals in tissues of cattle exposed to sludge-treated pastures for eight years. Am. J. Vet. Res. 46, 703–707 (1985). 3922265

[b34] Vreman K., Vanderveen N. G., Vandermolen E. J. & Deruig W. G. Transfer of cadmium, lead, mercury and arsenic from feed into milk and various tissues of dairy-cows - chemical and pathological data. Neth. J Agr. Sci. 34, 129–144 (1986).

[b35] Smith R. M. . Effects of long-term dietary cadmium chloride on tissue, milk, and urine mineral concentrations of lactating dairy cows. J. Dairy Sci. 69, 4088–4096 (1991). 10.2527/1991.69104088x 1778822

[b36] Vidovic M. M., Vidovic M. U. & Rodic M. N. Heavy metals in relation to soil-fodder-milk. http://www.Prague 2003.fsu. edu/content/pdf/606.pdf (2003).

[b37] Prister B. S. About behaviour of U in some components of biological chain. Dokl. VASHNIL 1, 31–33 (in Russian) (1967).

[b38] Martyshov V. Z., Burov N. I. & Antakova N. N. Uranium metabolism in farm animals. In second all union conference on agricultural radiology, **II**, 150–151 (1984).

[b39] Štrok M. & Smodiš B. Transfer of natural radionuclides from hay and silage to cow’s milk in the vicinity of a former uranium mine. J. Environ. Radioact. 110, 64–68 (2012). 22387974 10.1016/j.jenvrad.2012.02.009

[b40] Sirotkin A. N., Burov N. I., Tyumenov L. N. & Griskin A. I. On the behaviour of strontium-90, radiocaesium, cerium-144, ruthenium-106, antimony-125 and zirconium-95 in cattle. Radiobiologia 10, 629 (in Russian) (1970).

[b41] Sirotkin A. N., Grishin A. I. & Tyumenev L. N. Migration in the trophic chain and metabolism of radioactive products of neutron activation in farm animals. In Problems of animal radioecology (ed. Ilyenko A. I.) Nauka, 103–123 (in Russian) (1980).

[b42] Van Bruwaene R. . Metabolism of Cr-51, Mn-54, Fe-59 and Co-60 in lactating dairy cows. Health Phys. 46, 1069–1082 (1984). 6539319 10.1097/00004032-198405000-00007

[b43] Fesenko S. . Review of Russian language studies on radionuclide behaviour in agricultural animals: part 1. Gut absorption. J. Environ. Radioact. 98, 85–103 (2007a). 17728027 10.1016/j.jenvrad.2007.02.011

[b44] Fletcher C. R. The radiological hazard of zirconium-95 and niobium-95. Health Phys. 16, 209–220 (1969). 5772185 10.1097/00004032-196902000-00011

[b45] Shiraishi Y. & Ichikawa R. Absorption and retention of Ce-144 and Zr-95/Nb-95 in newborn juvenile and adult rats. Health Phys. 22, 373–378 (1972). 5045197 10.1097/00004032-197204000-00009

[b46] Coughtrey P. J. & Thorne M. Radionuclide distribution and transport in terrestrial and aquatic ecosystems. A critical review of data. Volume 1. Balkema, Rotterdam (1983).

[b47] Johnson J. E., Ward G. M., Ennis M. E. Jr. & Boamah K. N. Transfer coefficients of selected radionuclides to animal products. 1. Comparison of milk and meat from dairy cows and goats, Health Phys. 54, 161–166 (1988). 3338913 10.1097/00004032-198802000-00004

[b48] Fesenko S. . Review of Russian language studies on radionuclide behaviour in agricultural animals: part 2. Transfer to milk. J. Environ. Radioact. 98, 104–136 (2007b). 17766017 10.1016/j.jenvrad.2007.06.007

[b49] Gustafson G. M. Partitioning of nutrient and trace elements in feed among milk, faeces and urine by lactating dairy cows. Acta Agr. Scand. A-An 50, 111–120 (2000).

[b50] Herwig N. . Multi-element screening in milk and feed by SF-ICP-MS. Food Chem. 124, 1223–1230 (2011).

[b51] Kume S.-I. Effect of dietary trace element level and hot environmental temperature on trace element nutrition of Holstein dairy cattle 317-705. National Agricultural Experimental station, MAFF, Nishigoshi, Kumamoto, Japan (1989).

[b52] Kinkaid R. L. . Effect of dietary cobalt supplementation on cobalt metabolism and performance of dairy cattle. J. Dairy Sci. 86, 1405–1441 (2003). 12741565 10.3168/jds.S0022-0302(03)73724-2

[b53] Turtianen T., Kostianem E., Solatie D. & Rasilainen. Investigations of naturally occurring radionuclides in dairy cattle diet and milk. International Conference on Radioecology & Environmental Radioactivity Barcelona, Spain (2014).

[b54] Phipps R. H. . Selenium supplementation of lactating dairy cows: effects on milk production and total selenium content and speciation in blood, milk and cheese. Animal 2, 1610–1618 (2008). 22444012 10.1017/S175173110800298X

[b55] Juniper D. T., Phipps R. H., Jones A. K. & Bertin G. Selenium supplementation of lactating dairy cows: effect on selenium concentration in blood, milk, urine, and faeces. J. Dairy Sci. 89, 3544–3551 (2006). 16899690 10.3168/jds.S0022-0302(06)72394-3

